# Characteristics of Hydrodynamic Parameters of Different Understory Vegetation Patterns

**DOI:** 10.3390/plants14162556

**Published:** 2025-08-17

**Authors:** Chenhui Zhang, Jiali Wang, Jianbo Jia

**Affiliations:** 1 College of Soil and Water Conservation, Central South University of Forestry and Technology, Changsha 410004, China; zhangchenhui0707@163.com (C.Z.); 15200325900@163.com (J.W.); 2 Hunan Lutou Forest Ecosystem Orientation Observation and Research Station, Yueyang 410004, China

**Keywords:** hydrodynamic parameter, vegetation quilt configuration mode, soil erosion, simulated rainfall in the field, sediment yield

## Abstract

The presence of understory vegetation not only influences slope-scale soil and water conservation but also exerts a profound effect on hydrodynamic characteristics and the processes of runoff and sediment production. Therefore, in this study, different vegetation types and vegetation coverages (bare land, 30%, 60%, and 90%) were set up by simulating rainfall (45, 60, 90, and 120 mm·h^−1^) to evaluate the runoff-sediment process and the response characteristics of hydrodynamic parameters. The results showed that increasing vegetation cover significantly reduced soil erosion on forest slopes (*p* < 0.05). When the vegetation cover ranged from 60% to 90%, vegetation pattern C and pattern D were the most effective in suppressing erosion, where increased cover improved runoff stability. Under low-cover conditions, overland flow tended toward turbulent and rapid regimes, whereas under high cover conditions, flow was primarily laminar and slow. Patterns C and D significantly reduced flow velocity and water depth (*p* < 0.05). Structural equation patterning revealed that, under different vegetation patterns, the runoff power (*ω*), Reynolds number (*Re*), and resistance coefficient (*f*) more effectively characterized the erosion process. Among these, the Reynolds number and runoff power were the dominant factors driving erosion on red soil slopes. By contrast, runoff shear stress was significantly reduced under high-cover conditions and showed weak correlation with sediment yield, suggesting that it was unsuitable as an indicator of slope erosion. Segmental vegetation arrangements and increasing vegetation cover near runoff outlets—especially at 60–90% coverage—effectively reduced soil erosion. These findings provide scientific insight into the hydrodynamic mechanisms of vegetation cover on slopes and offer theoretical support for optimizing soil and water conservation strategies on hilly terrain.

## 1. Introduction

Vegetation restoration plays a vital role in the management of soil erosion [1,2,3]. Since 1999, the Chinese government has enforced extensive vegetation restoration. In the past 20 years, the total area of soil erosion in the red soil region of southern China has decreased by 64,800 km^2^ [4]. As one of the pioneer tree species to control soil erosion and economic value, Cunninghamia lanceolata is widely planted in the red soil region of southern China. This measure has achieved great results in inhibiting soil erosion. However, due to poor soil fertility and acidification, strong rainfall erosion capacity, and lack of understory vegetation, there is still moderate or even bad soil erosion in this area, resulting in new “soil erosion under forest” phenomena [5,6]. This phenomenon is often described as “green from a distance, yellow with flowing water up close.” In evaluating the contributions of various measures to soil erosion control, Zuazo et al. [7] consistently argued that ground cover is more important than canopy cover. Thus, controlling soil erosion in forests and improving forest soil quality have become the main objectives of soil and water loss control in the red soil area of southern China.

Understory vegetation plays a crucial role in controlling soil erosion in forests [8,9]. Although the canopy and forest leaves intercept most of the rainfall, in artificial forests without understory vegetation, the remaining raindrop splash can generate higher kinetic energy, leading to more soil detachment [10,11]. Understory vegetation effectively reduces the raindrop splash effect [10,12], increases flow resistance on slopes, and slows down the flow velocity [13]. Vegetation cover, as an economical and environmentally friendly soil and water conservation measure, has received widespread attention and recognition for its role in soil erosion prevention and control [14]. Vegetation coverage effectively weakens raindrop kinetic energy and increases surface roughness and soil aggregate stability [15,16]. By avoiding the direct impact of rainwater on the surface, it reduces direct exposure, thereby reducing soil particle movement. Zhang et al. [16] found that when the vegetation coverage reached 60%, it could effectively slow down the runoff rate, prevent soil erosion, and significantly improve soil stability. In addition, Zhu et al. [17] found that when the vegetation coverage was between 60% and 80%, the inhibition effect was the most significant through artificial rainfall experiments. However, the study by Deyi et al. [18] showed that only when the coverage rate reached 91.77% could it effectively play the role of soil and water conservation in the process of erosion. An increase in vegetation cover can effectively suppress soil erosion, but little is known about the reasonable vegetation cover under forests.

Vegetation coverage can effectively inhibit runoff and sediment production, changing the hydrodynamic parameters of the slope [19]. This process not only optimizes the movement characteristics of slope water flow but also enhances soil stability, thereby slowing down the occurrence of soil erosion. Some scholars have also found that increasing vegetation coverage can effectively improve slope erosion resistance [6]. Vegetation reduces flow velocity, increases resistance coefficients, and improves hydrodynamic characteristics, thus enhancing the erosion resistance of slope soil and the hindrance effect on surface runoff. Under high cover conditions, the decrease in runoff velocity reduces the intensity of erosion on slope hydrodynamic parameters [19,20]. Studies have demonstrated that vegetation cover measures on yellow soil slopes can reduce flow velocity, water depth, and runoff shear stress, thereby mitigating erosion [21]. Léonard et al. [22] and Peiqing et al. [23], through calculations of critical runoff shear stress and critical water flow power, found that the critical unit stream power values were 0.0127 m/s for vegetation plots and 0.0169 m/s for shrub plots, demonstrating the beneficial soil and water conservation effects of vegetation cover.

In recent years, scholars both domestically and internationally have conducted in-depth studies on the relationship between vegetation and soil erosion [6,16,24,25,26]. Research has shown that vegetation has a significant effect in preventing soil erosion; however, a complex linear relationship exists between vegetation cover and sediment interception. When vegetation cover reaches a certain threshold, the additional benefits in reducing sediment loss become less pronounced [6]. Vegetation pattern is a key factor influencing surface runoff generation and sediment yield [24]. El Kateb et al. [25] explored the impact of vegetation cover patterns on slope erosion through field experiments. Zhang et al. [16] conducted flume experiments to study the effect of varying slope gradients and vegetation strip positioning on runoff and sediment yield, with the results showing that different positions of vegetation strips significantly altered the temporal and spatial variation in slope flow velocity, and the runoff-sediment characteristics were closely related to the distribution of slope flow velocity. Zhang et al. [26], based on simulated rainfall experiments, found that compared to bare slopes, four patchy distributions of Artemisia (horizontal vegetation strips, vertical vegetation strips, checkerboard, and “X”-shaped) reduced the average flow velocity by 14.1% to 60.5%. Under rainfall intensities of 60 mm·h^−1^ and 90 mm·h^−1^, these four vegetation cover distributions exhibited the best performance in reducing flow velocity. Furthermore, Sun et al. [20] conducted field rainfall experiments and found that vegetation cover with poor hydrological connectivity showed a stronger sediment interception capability compared to vegetation cover with good hydrological connectivity. For example, vegetation with 40% cover in horizontal, random, and S-shaped paths reduced the sediment content by 89.9%, 93.5%, and 50.6%, respectively, compared to bare slopes. This indicated that different vegetation cover distribution patterns significantly affect runoff and sediment yield on slopes. However, the quantitative study of vegetation cover patterns has not yet been resolved. Therefore, it is necessary to establish a coupled relationship between the quantitative indicators of different vegetation cover patterns and slope runoff-sediment yield.

Although previous studies have revealed the effects of vegetation cover on soil erosion and hydrodynamics, there are still some gaps in the current research. Therefore, this study focuses on red soil slopes and uses field simulated rainfall experiments to investigate the effects of different understory vegetation coverages and patterns on soil erosion and their hydrodynamic response mechanisms. The aim is to provide a scientific theoretical basis for soil and water conservation practices and vegetation restoration in the southern red soil region by examining the relationship between understory vegetation cover, vegetation pattern, runoff, sediment yield, and hydrodynamic parameters.

## 2. Materials and Methods

### 2.1. Study Area

**Figure 1:**
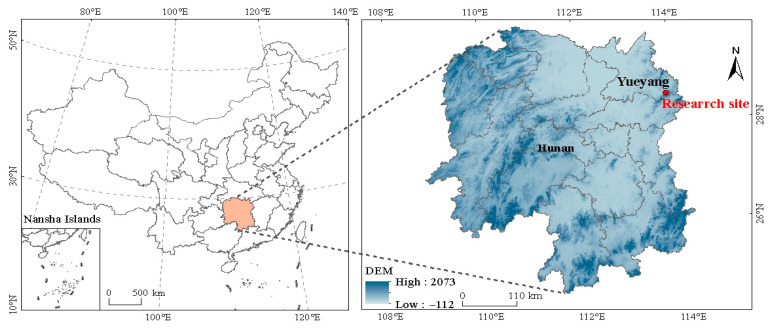
Study area.

The experiment was located in the National Observation and Research Station of the Lutou Forest Ecosystem in Hunan Province (28°31′7″–28°38′ N, 113°51′52″–113°58′24″ E) (Figure 1). The region is situated in a humid continental monsoon climate zone, transitioning from the mid-subtropical to the northern subtropical climate belt. The climate in this area is distinct, with four distinct seasons. The average annual sunshine duration is 1930 h, and the average air humidity is 82%, providing favorable light conditions and abundant precipitation, with an annual average rainfall of 1450.8 mm. The main rainy season occurs from March to July, accounting for 66.8% of the total annual precipitation. The annual average temperature of the forest is 15.89 °C. The dominant soil types in the area include red soil, mountain yellow soil, and mountain yellow-brown soil, with a diverse range of parent material for soil formation.

### 2.2. Experiment Design

**Figure 2:**
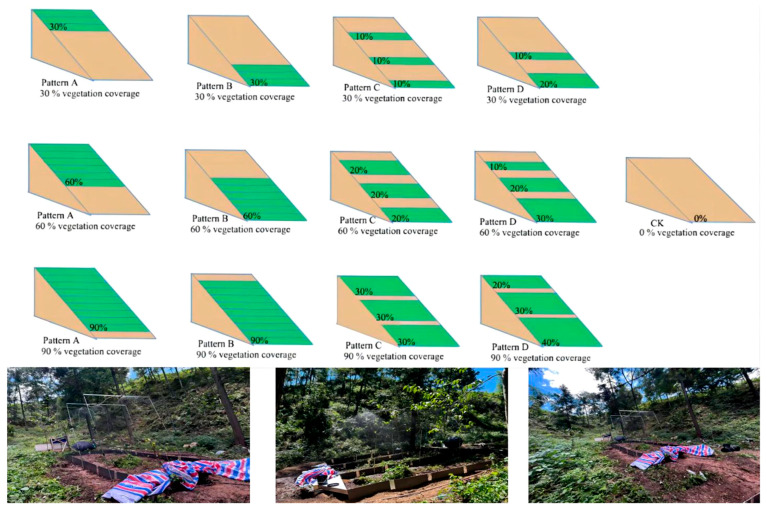
Schematic diagram of vegetation layout.

The experiment was conducted in July 2024, with a total of 10 runoff plots set up. These included: pattern A, where vegetation was primarily concentrated at the runoff outlet (30%, 60%, and 90% cover); pattern B, where vegetation was located uphill of the runoff plots (30%, 60%, and 90% cover); pattern C, with vegetation was evenly distributed across the upper, middle, and lower slopes (30%, 60%, and 90% cover); pattern D, where vegetation coverage gradually increased by 10% downslope (30%, 60%, and 90% cover); a control plot (0% cover); and plots with 30%, 60%, and 90% vegetation cover (Figure 2). To investigate the effect of different vegetation coverages on slope erosion and sediment yield, artificial methods were used to control the vegetation cover. The vegetation cover was evenly distributed along the slope using a design created in Photoshop for the spatial arrangement of vegetation, which was then applied to the slope surface. A digital camera was used to take photos at 2 m above the slope, and Photoshop and ArcGIS 10.2 were used for pixel analysis. The actual vegetation coverage was determined by looking at the area of the green shadow part. Based on the calculated results, the vegetation cover was adjusted until the difference between the actual and designed cover was within 3%, at which point the experiment began (Figure 2). The vegetation selected for the slope surface was mainly Houttuynia cordata (*Houttuynia cordata* Thunb.), which is suitable for growth in the region. The slope in each plot had an angle of approximately 30°, with a slope length of 5 m and width of 2 m. PVC boards were used as slope boundaries, buried 30 cm underground and protruding 20 cm above the ground to prevent soil particle dispersion caused by raindrop splashing during artificial rainfall. A collection trough was set up at the slope outlet and connected to a runoff bucket to collect runoff and sediment generated during the artificial rainfall experiments.

The experiment used full-jet standard and wide-angle solid cone nozzles. Simulated rainfall experiments were carried out under the forest. The simulated rainfall system consisted of a pumping pump, variable booster pump, connecting pipe, nozzle, and bracket. Rainfall strength was controlled by regulating the booster pump water pressure and replacing the nozzle. According to the long-term rainfall intensity data from May to September in this area, this study selected rainfall intensities with high frequency in this area: 45, 60, 90, and 120 mm·h^−1^. The pre-experiment found that the runoff was stable within 15 min under the rainfall intensity of 120 mm·h^−1^, so the rainfall time was selected as 30 min. There were a total of 16 experimental combinations, and each combination was repeated three times (three repeated rainfall events did not show significant differences), resulting in a total of 192 rainfall events. For downward spraying nozzles with initial velocity, when the rainfall height was between 2 and 3 m, raindrops of different diameters could reach a terminal velocity of 2 to 2.9 m·s^−1^. The characteristics of the simulated rainfall were measured by physical and numerical simulations and compared with natural rainfall conditions. The raindrop size was about 1 mm to 5 mm, the corresponding terminal velocity was 4.76 m·s^−1^ to 10.64 m·s^−1^, the impact velocity was 5.56 m·s^−1^ to 9.63 m·s^−1^, and the kinetic energy was 0.0081 mJ to 3.0342 mJ. When the rainfall height is 2–3 m, simulated rainfall experiments are very close to natural rainfall [27]. Therefore, in this experiment, the rainfall height was designed to be 3 m, and after actual measurement, the rainfall uniformity exceeded 85%. The site conditions of all experimental plots were basically similar.

### 2.3. The Test Process

Prior to each rainfall experiment, the rainfall intensity was calibrated to meet the experimental design requirements. In addition, to ensure consistent initial soil moisture content on the slope surface, a pre-rainfall treatment was conducted. The pre-rainfall intensity was set at 45 mm·h^−1^ and was stopped immediately when runoff began. After the pre-rainfall treatment, the slope surface was covered with a protective layer and left undisturbed for 24 h before conducting the formal simulated rainfall experiment. The rainfall duration for each simulated rainfall event was set to 30 min after runoff initiation. During each rainfall event, the runoff time was recorded, and runoff samples were collected once every minute for the first 10 min after runoff began. After 10 min, samples were collected every 4 min, for a total of 15 samples per rainfall event. At the end of the experiment, a measuring cylinder was used to measure the total runoff volume of each sample, in milliliters (mL). The samples were then left to settle for 24 h. The upper clear liquid was separated, the remaining sediment was transferred to an aluminum container, and the supernatant sediment sample was taken out and transferred to the aluminum metal box (volume = 20 cm × 10 cm × 8 cm) by washing the bottle to accelerate the drying of the sample. The weight of the aluminum box was weighed before the transfer, and the weight of the sediment = the total weight of the drying − the weight of the aluminum box. After drying in an oven at 105 °C for 24 h, the sediment was weighed, and the weight recorded represented the total sediment yield, which was considered the soil erosion amount, in grams (g).

The runoff velocity on the slope surface was measured using the tracer dye method (KMnO_4_). Measurements were conducted at three locations: the upper, middle, and lower sections of the slope. The length of the velocity measurement zone was 0.5 m, and each measurement was repeated three times to obtain the average value, which represented the average surface flow velocity. The temperature of the water flow was also recorded. Three equidistant cross-sections were selected on the slope, and the flow width at each cross-section was measured. The average value of these measurements was taken as the flow width for the entire slope. Based on the measured flow width, velocity, and other data, the hydrodynamic parameters were subsequently calculated.

### 2.4. Indicator Calculation

The calculation methods for the slope surface flow hydrodynamic parameters are as follows [28,29]:

(1) The equation for calculating the average velocity of runoff is:

(1) $$u=ku_{0}$$

In the equation, *u* is the average flow velocity of slope (mm·s^−1^); *u*_0_ is the surface flow velocity (mm·s^−1^); and *k* is the correction coefficient, which is 0.75 in this paper.

(2) The runoff depth calculation equation is:

(2) $$h=\frac{Q}{u\cdot B\cdot t}$$

In the equation, *h* is the slope water depth (mm), *Q* is the total runoff in the rainfall duration *t* (m^3^), and *B* is the width of the water passage section (m).

(3) The Reynolds number is calculated using the following equation:

(3) $$R_{e}=\frac{uR}{v}$$

In the equation, *R* is the hydraulic radius (m), which, for slope flow, is approximately equal to the average water depth (m) during that period; and *v* is the kinematic viscosity of water (m^2^·s), where *v* = 0.01775/(1 + 0.0337*t* + 0.000221*t*^2^).

(4) The Froude number is calculated using the following equation:

(4) $$F_{r}=\frac{u}{\sqrt{gh}}$$

In the equation, *g* is the gravitational acceleration (m·s^−2^), with a value of 9.8.

(5) The calculation equation of Darcy–Weisbach resistance coefficient is:

(5) $$f=\frac{8gRJ}{u^{2}}$$

In the equation, *J* is the hydraulic energy slope. When the slope is small, it can be replaced by the sine value of the slope, that is, *J* = sinα, where α is the slope.

(6) The calculation equation of Manning roughness coefficient is:

(6) $$n=\frac{R^{\frac{2}{3}}\times J^{\frac{1}{2}}}{u}$$

In the equation, *R* is the hydraulic radius (m), and *J* is the hydraulic energy.

(7) The runoff shear stress is calculated using the following equation:

(7) τ=γ⋅R⋅J

In the equation, *τ* is the runoff shear stress (Pa), and *γ* is the unit weight of water (kg·m^−3^).

(8) The runoff power is calculated using the following equation:

(8) ω=τ⋅u

In the equation, *ω* is the runoff power (N/(m·s)).

### 2.5. Data Analysis and Plotting Tools

The experimental data were organized and calculated using Excel 2021. Data analysis, including statistical tests and correlation analysis, was performed using SPSS 27.0. Graphs and charts were created using Origin 2024 for visualization, ensuring the accuracy of data processing and the clarity of result presentation.

## 3. Results and Analysis

### 3.1. Characteristics of Runoff and Sediment Yield on Slopes Under Different Understory Vegetation Patterns

#### 3.1.1. Characteristics of Slope Runoff Under Different Understory Vegetation Patterns

**Figure 3:**
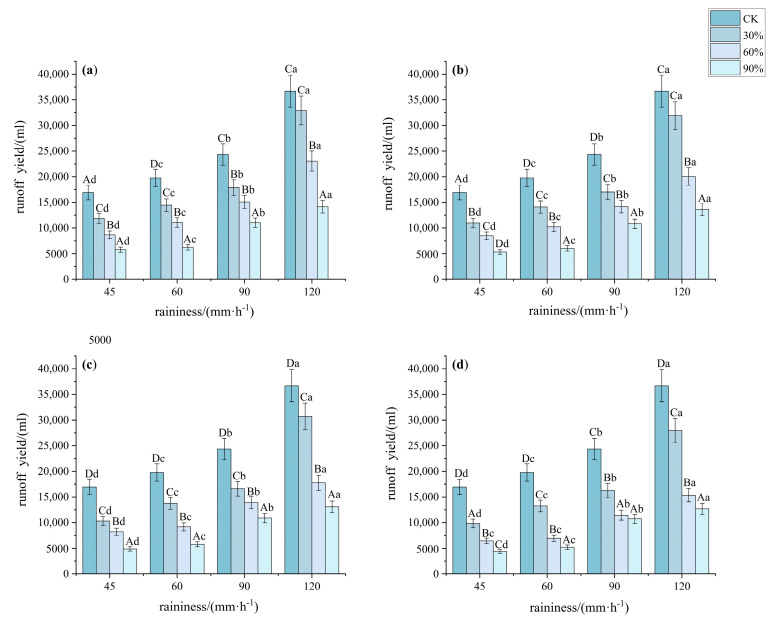
Characteristics of runoff under different understory vegetation patterns. Note: The significance of differences under the same rainfall intensity but at bare land, 30%, 60%, and 90% cover is indicated using uppercase letters (A, B, C, D). (**a**) Pattern A; (**b**) Pattern B; (**c**) Pattern C; (**d**) Pattern D. The significance of differences at the same vegetation cover but under different rainfall intensities is indicated using lowercase letters (a, b, c, d) (significance level *p* < 0.05), The research used Turkey’s test under one-way analysis of variance for data with homogeneous variance. When the variance is not homogeneous, the Welch test (corrected F test) was used. The error bar represents the standard error (SD).

The results are shown in Figure 3. Significant differences in runoff were observed under bare land, 30%, 60%, and 90% conditions (*p* < 0.05, *F* > 300). Runoff decreased as vegetation cover increased: bare land > 30% > 60% > 90%. Higher vegetation cover led to lower cumulative runoff. Under a rainfall intensity of 45 mm·h^−1^, the runoff at 30%, 60%, and 90% cover was 63.45%, 46.99%, and 30.07% of that from bare land, respectively. The runoff for patterns A, B, C, and D was 51.83%, 48.82%, 46.09%, and 40.61% of the runoff from bare land, respectively. Under a rainfall intensity of 60 mm·h^−1^, the runoff at 30%, 60%, and 90% cover was 70.32%, 47.30%, and 29.42% of that from bare land, respectively. The runoff for patterns A, B, C, and D was 53.57%, 51.17%, 48.48%, and 42.83% of the runoff from bare land, respectively. Under a rainfall intensity of 90 mm·h^−1^, the runoff at 30% cover was significantly higher than that at the other two levels of vegetation cover, while pattern D had significantly lower runoff than the other patterns (*p* < 0.05, *F* > 600). Under a rainfall intensity of 120 mm·h^−1^, the runoff reduction rate at 30% cover was significantly lower than that at other rainfall intensities, and 90% cover reduced runoff more effectively than the other patterns (*p* < 0.05, *F* > 1100).

#### 3.1.2. Characteristics of Slope Sediment Yield Under Different Understory Vegetation Patterns

**Figure 4:**
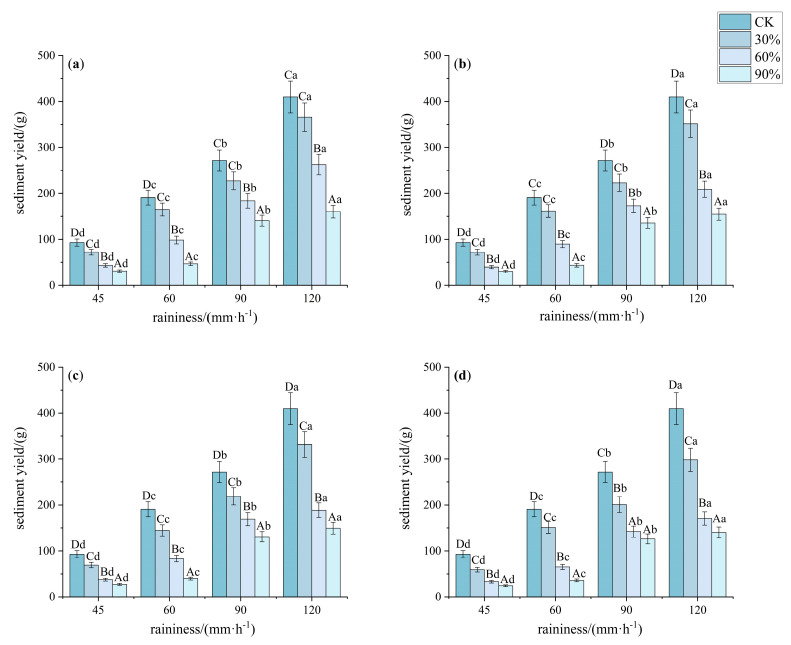
Characteristics of sediment yield under different understory vegetation patterns. Note: The significance of differences under the same rainfall intensity but at bare land, 30%, 60%, and 90% cover is indicated using uppercase letters (A, B, C, D). (**a**) Pattern A; (**b**) Pattern B; (**c**) Pattern C; (**d**) Pattern D. The significance of differences at the same vegetation cover but under different rainfall intensities is indicated using lowercase letters (a, b, c, d) (significance level *p* < 0.05), The research used Turkey’s test under one-way analysis of variance for data with homogeneous variance. When the variance is not homogeneous, the Welch test (corrected F test) was used. The error bar represents the standard error (SD).

The sediment yield process under bare land, 30%, 60%, and 90% conditions was similar to the runoff process. As vegetation cover increased, the cumulative sediment yield changed as follows: bare land > 30% > 60% > 90%. Under a rainfall intensity of 45 mm·h^−1^, the sediment yield at 30%, 60%, and 90% cover was 73.33%, 41.32%, and 30.21% of that from bare land, respectively. Pattern D had significantly lower sediment yield than the other patterns (*p* < 0.05, *F* > 900), with the cumulative sediment yield being 52.56%, 50.89%, 47.93%, and 41.64% of that from bare land for patterns A, B, C, and D, respectively. Under a rainfall intensity of 60 mm·h^−1^, the sediment yield at 30%, 60%, and 90% cover was 81.52%, 44.08%, and 21.68% of that from bare land, respectively. The cumulative sediment yield from pattern D was significantly lower than that from patterns A, B, and C (*p* < 0.05, *F* > 900). Under rainfall intensities of 90 mm·h^−1^ and 120 mm·h^−1^, when the vegetation cover exceeded 60%, the sediment yield did not increase significantly with higher cover (*p* < 0.05, *F* > 1300). However, the cumulative sediment yield followed the order: pattern A > pattern B > pattern C > pattern D (Figure 4).

### 3.2. Characteristics of Slope Hydraulic Parameters Under Different Understory Vegetation Patterns

#### 3.2.1. Average Flow Velocity and Average Runoff Depth of Slope Flow

**Figure 5:**
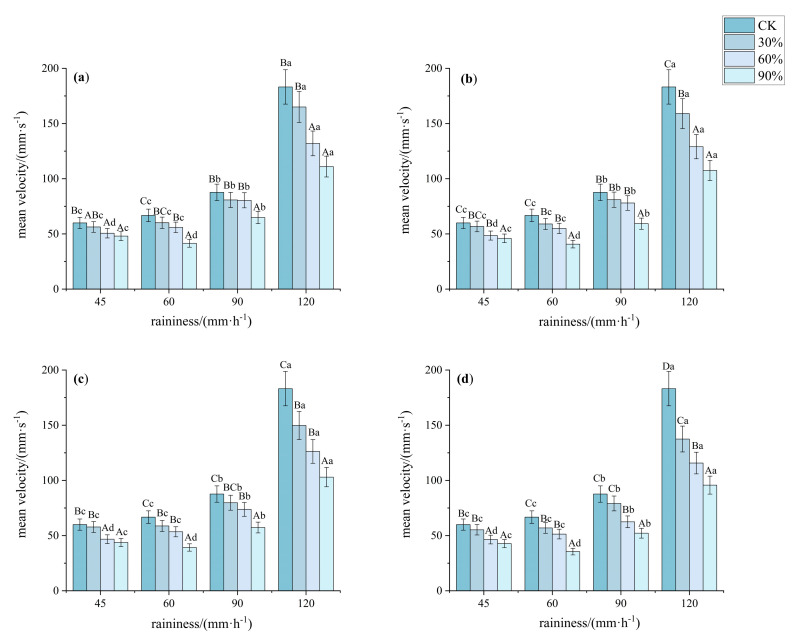
Characteristics of average flow velocity under different understory vegetation patterns. Note: The significance of differences under the same rainfall intensity but at bare land, 30%, 60%, and 90% cover is indicated using uppercase letters (A, B, C, D). (**a**) Pattern A; (**b**) Pattern B; (**c**) Pattern C; (**d**) Pattern D. The significance of differences at the same vegetation cover but under different rainfall intensities is indicated using lowercase letters (a, b, c, d) (significance level *p* < 0.05), The research used Turkey’s test under one-way analysis of variance for data with homogeneous variance. When the variance is not homogeneous, the Welch test (corrected F test) was used. The error bar represents the standard error (SD).

**Figure 6:**
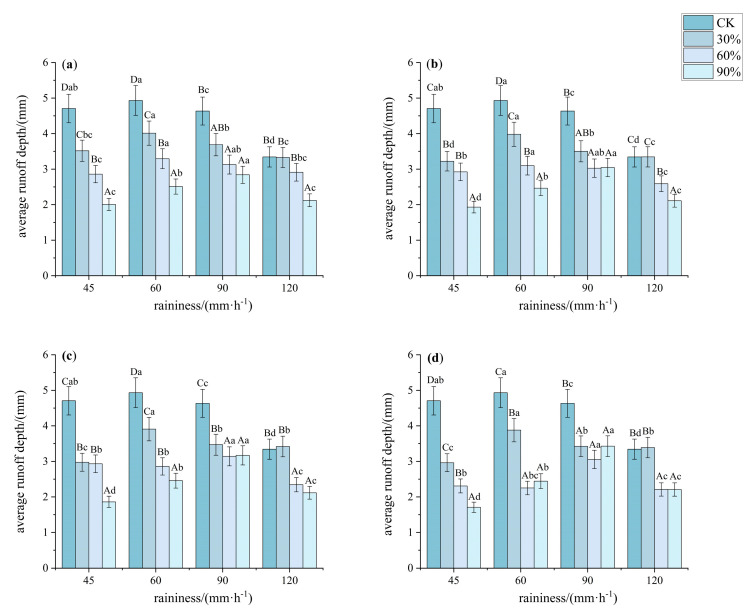
Characteristics of average runoff depth under different understory vegetation patterns. Note: The significance of differences under the same rainfall intensity but at bare land, 30%, 60%, and 90% cover is indicated using uppercase letters (A, B, C, D). (**a**) Pattern A; (**b**) Pattern B; (**c**) Pattern C; (**d**) Pattern D. The significance of differences at the same vegetation cover but under different rainfall intensities is indicated using lowercase letters (a, b, c, d) (significance level *p* < 0.05), The research used Turkey’s test under one-way analysis of variance for data with homogeneous variance. When the variance is not homogeneous, the Welch test (corrected F test) was used. The error bar represents the standard error (SD).

Vegetation cover significantly regulated rainfall erosion on steep red soil slopes, which exhibited differentiated characteristics under different rainfall intensities (Figure 5 and Figure 6). As rainfall intensity increased, surface flow velocity rose, reaching 35.5 to 183.16 mm·s^−1^ at 120 mm·h^−1^. The average flow velocity on bare land slopes was significantly higher than that at other coverages (*p* < 0.05, *F* > 350). Under different rainfall intensities and vegetation cover conditions, the slope runoff depth also showed significant differences (*p* < 0.05), with rainfall intensity significantly increasing the average flow velocity (*p* < 0.05). Specifically, under a rainfall intensity of 45 mm·h^−1^, the average flow velocities at 30%, 60%, and 90% cover were 94.31%, 80.19%, and 75.28% of that on bare land, respectively. The average flow velocities for patterns A, B, C, and D were 86.14%, 84.11%, 82.49%, and 80.32% of the bare land velocity, respectively. Under rainfall intensities of 60 and 90 mm·h^−1^, the flow velocities at 90% cover were significantly lower than those at other coverages, while there were no significant differences in the average flow velocities between 30% and 60% cover (*p* < 0.05, *F* > 480). Additionally, there were no significant differences in the average flow velocities among the four patterns (*p* < 0.05, *F* > 1000). Under a rainfall intensity of 120 mm·h^−1^, the average flow velocities at 60% and 90% cover were significantly lower than that at 30% cover, and when the vegetation cover reached 60%, the reduction in average flow velocity was not significant (*p* < 0.05, *F* > 350). Pattern D and pattern C showed significantly lower flow velocities compared to the other two patterns (*p* < 0.05, *F* > 350).

#### 3.2.2. Reynolds Number and Froude Number

**Figure 7:**
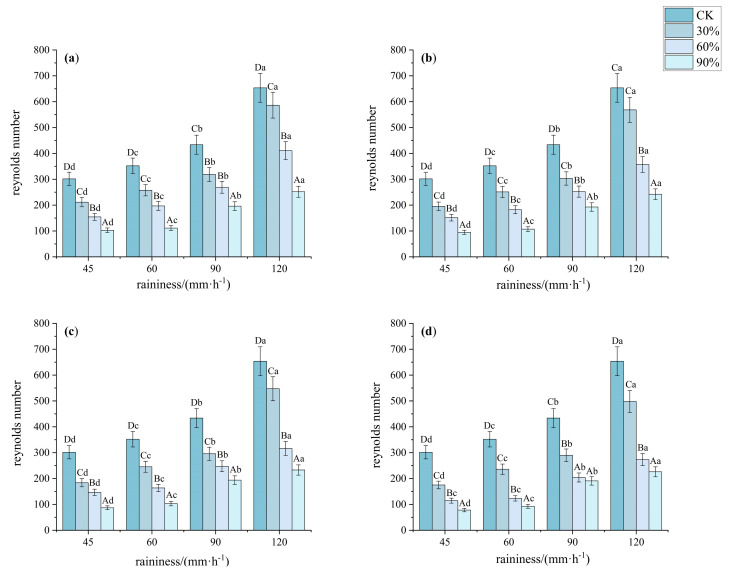
Characteristics of Reynolds Number under different understory vegetation patterns. Note: The significance of differences under the same rainfall intensity but at bare land, 30%, 60%, and 90% cover is indicated using uppercase letters (A, B, C, D). (**a**) Pattern A; (**b**) Pattern B; (**c**) Pattern C; (**d**) Pattern D. The significance of differences at the same vegetation cover but under different rainfall intensities is indicated using lowercase letters (a, b, c, d) (significance level *p* < 0.05), The research used Turkey’s test under one-way analysis of variance for data with homogeneous variance. When the variance is not homogeneous, the Welch test (corrected F test) was used. The error bar represents the standard error (SD).

The Reynolds number (*Re*) is an important dimensionless number in fluid dynamics that characterizes the relative interaction between inertial forces and viscous forces. In this study, as an indicator of the turbulence level of fluid flow, the Re values were less than 500 for all runoff durations, except under a rainfall intensity of 120 mm·h^−1^, where CK and 30% vegetation cover exceeded 500 (Figure 7). This indicated that slope runoff is laminar, and the *Re* value decreases with increasing vegetation cover. According to the data in the table, the Reynolds number significantly decreased at 90% cover (*p* < 0.05, *F* > 300), with patterns C and D showing significantly lower values than the other patterns. Additionally, under bare land and low-coverage conditions, the Reynolds number rapidly increased with rainfall intensity, making the flow more likely to transition to turbulent flow, thereby increasing the risk of erosion. However, under high-cover conditions, the change in Reynolds number with increasing rainfall intensity was not significant, demonstrating the significant dissipative effect of vegetation on rainfall energy.

**Figure 8:**
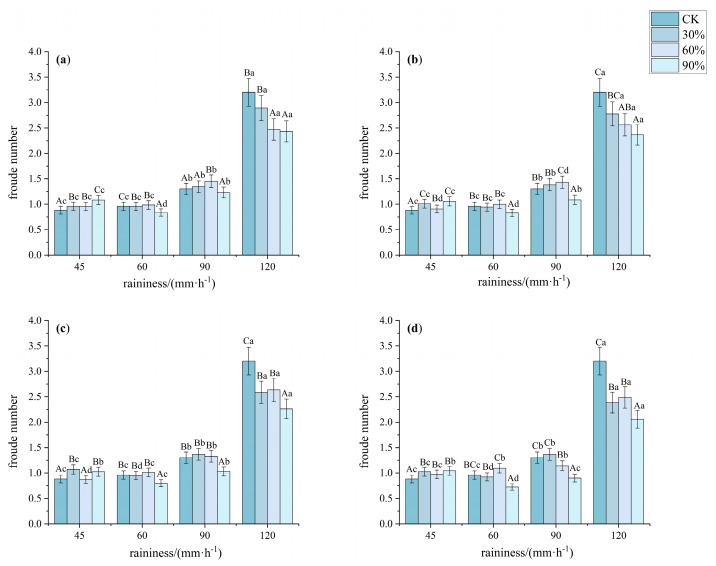
Characteristics of Froude number under different understory vegetation patterns. Note: The significance of differences under the same rainfall intensity but at bare land, 30%, 60%, and 90% cover is indicated using uppercase letters (A, B, C, D). (**a**) Pattern A; (**b**) Pattern B; (**c**) Pattern C; (**d**) Pattern D. The significance of differences at the same vegetation cover but under different rainfall intensities is indicated using lowercase letters (a, b, c, d) (significance level *p* < 0.05), The research used Turkey’s test under one-way analysis of variance for data with homogeneous variance. When the variance is not homogeneous, the Welch test (corrected F test) was used. The error bar represents the standard error (SD).

The Froude number (*Fr*) characterizes the ratio between the inertial forces and gravitational effects of the flow. It can be seen from Figure 8 that as vegetation cover increases, the Froude number first increases and then decreases, indicating a transition from rapid flow to slow flow. At low coverage (30%), the Froude number was significantly higher than that at other coverages, particularly reaching 3.23 under a rainfall intensity of 120 mm·h^−1^, with the runoff flow tending toward rapid flow. As the vegetation cover increased to 60%, the Froude number significantly decreased. At 90% cover, even when the rainfall intensity reached 120 mm·h^−1^, the Froude number was still only 0.75.

### 3.3. Characteristics of Slope Dynamic Parameters of Different Understory Vegetation Patterns

**Figure 9:**
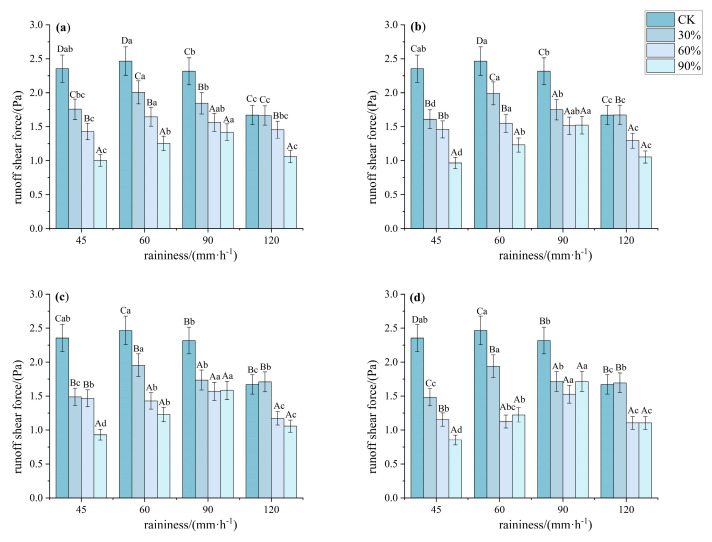
Characteristics of runoff shear force under different understory vegetation patterns. Note: The significance of differences under the same rainfall intensity but at bare land, 30%, 60%, and 90% cover is indicated using uppercase letters (A, B, C, D). (**a**) Pattern A; (**b**) Pattern B; (**c**) Pattern C; (**d**) Pattern D. The significance of differences at the same vegetation cover but under different rainfall intensities is indicated using lowercase letters (a, b, c, d) (significance level *p* < 0.05), The research used Turkey’s test under one-way analysis of variance for data with homogeneous variance. When the variance is not homogeneous, the Welch test (corrected F test) was used. The error bar represents the standard error (SD).

As shown in Figure 9, the runoff shear stress under bare land, 30%, 60%, 90%, and rainfall intensity conditions exhibited significant differences (*p* < 0.05). The hydrodynamic mechanism was primarily determined by the vegetation’s regulation of surface roughness and its ability to dissipate raindrop kinetic energy. Specifically, under a rainfall intensity of 45 mm·h^−1^, the runoff shear stress at 30%, 60%, and 90% cover was 67.33%, 58.53%, and 39.88% of that on bare land, respectively. The runoff shear stress for Patterns A, B, C, and D was 59.36%, 57.20%, 55.01%, and 49.42% of that on bare land, respectively. Under a rainfall intensity of 60 mm·h^−1^, the runoff shear stress at 90% cover was significantly lower than at other coverages, and pattern D had significantly lower runoff shear stress compared to the other patterns (*p* < 0.05, *F* > 10). Under rainfall intensities of 90 and 120 mm·h^−1^, there were no significant differences in runoff shear stress between 60% and 90% cover, while pattern C and pattern D showed significantly lower shear stress compared to the other two patterns (*p* < 0.05, *F* > 6).

**Figure 10:**
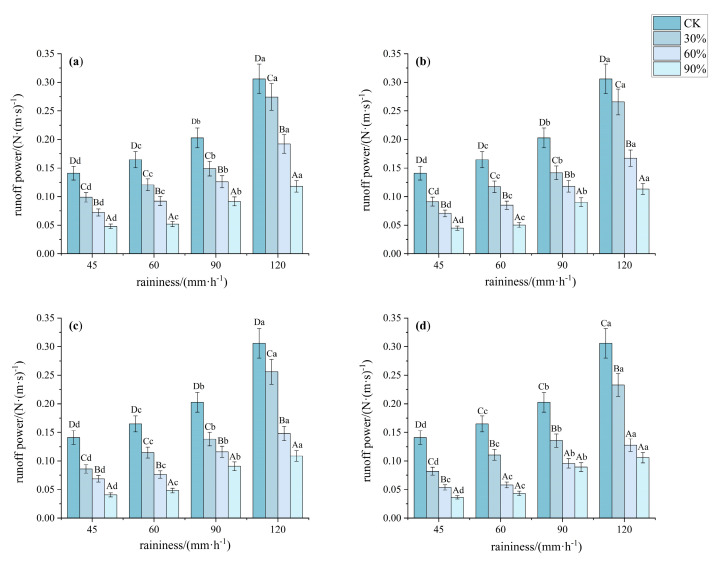
Characteristics of runoff power under different understory vegetation patterns. Note: The significance of differences under the same rainfall intensity but at bare land, 30%, 60%, and 90% cover is indicated using uppercase letters (A, B, C, D). (**a**) Pattern A; (**b**) Pattern B; (**c**) Pattern C; (**d**) Pattern D. The significance of differences at the same vegetation cover but under different rainfall intensities is indicated using lowercase letters (a, b, c, d) (significance level *p* < 0.05), The research used Turkey’s test under one-way analysis of variance for data with homogeneous variance. When the variance is not homogeneous, the Welch test (corrected F test) was used. The error bar represents the standard error (SD).

Based on the statistical analysis of runoff power, as the vegetation cover increased under the same rainfall intensity, the runoff power decreased (Figure 10). Under rainfall intensities of 90 and 120 mm·h^−1^, the runoff power at 90% vegetation cover was significantly lower than that at other coverages (*p* < 0.05, *F* > 6), indicating a weakening of the erosion effect. Under rainfall intensities of 45 and 90 mm·h^−1^, there were no significant differences in runoff power between 60% and 90% cover, but both were significantly lower than at 30% cover. Additionally, pattern D showed significantly lower runoff power compared to the other patterns (*p* < 0.05, *F* > 10). Similar results were observed under other rainfall intensities.

## 4. Discussion

### 4.1. Effects of Different Understory Vegetation Patterns on Runoff and Sediment Yield on Slopes

As vegetation cover increases, both slope runoff and sediment yield significantly decrease. The effectiveness of vegetation cover is attributed to its ability to intercept runoff. Under high-cover conditions, the uniform distribution of vegetation reduces the exposed soil area, fundamentally weakening the erosive capacity of water flow on the soil surface, This conclusion was confirmed in previous studies [6,25,30]. Under low coverage (30%), there is a larger area of exposed soil, and raindrops directly impact the surface, forming runoff, with a clear concentration effect of the water flow, leading to increased runoff and sediment yield [30]. Additionally, the sensitivity of runoff and sediment yield to rainfall intensity is higher under low cover. This is due to the loose soil structure of red soil slopes, which has weaker resistance to erosion [6,24]. Sun et al. [31] demonstrated that the cover layer reduces the Reynolds number (*Re*) and the level of soil disturbance caused by overland flow, thereby decreasing sediment yield. Omidvar et al. [32] also found that soil’s sensitivity to erosion decreases with increasing vegetation cover, leading to reduced surface flow velocity and increased runoff meandering, which ultimately lowers runoff and sediment yield. Similar results were obtained in this study.

The spatial distribution of vegetation is an important factor influencing the connectivity pathways of runoff and sediment [33,34]. Vegetation distribution patterns directly affect the hydrodynamic mechanisms of sediment yield, effectively reducing runoff velocity and thereby achieving better soil and water conservation [19,35]. Research by Ma et al. [36] showed that vegetation closer to the upper slope provides better functional connectivity, with faster runoff velocity and stronger erosion and sediment transport capacity. In our study, similar results were obtained, with the progressively increasing segmented interception pattern contributing more to reducing runoff and sediment than the other three patterns. This is likely because this pattern hinders the runoff, sediment generation, and transport processes on the slope, intercepts sediment in segments, reduces the kinetic and potential energy of the water flow, and weakens soil erosion, thus reducing both runoff and sediment yield [15,36].

### 4.2. Effects of Different Understory Vegetation Patterns on Hydrodynamic Parameters of Overland Flow

As vegetation cover increases, surface roughness, runoff interception, flow velocity reduction, and decreases in kinetic energy and water depth fluctuations effectively reduce erosion risk. Increased vegetation cover enhances surface roughness, slows down water flow, weakens runoff kinetic energy, and lowers erosion risk [15,28]. Different levels of vegetation cover significantly affect hydrodynamic parameters such as the Reynolds number, Froude number, runoff shear stress, and runoff power. Under a rainfall intensity of 120 mm·h^−1^, the Reynolds number and Froude number at 90% cover decreased by 70% and 76%, respectively, compared to 30% cover, indicating that high vegetation cover reduces runoff turbulence, thus weakening the erosive capacity of runoff on soil particles [37]. Unlike previous studies, runoff shear stress reached its maximum at 60% cover, possibly due to the alternating distribution of vegetation and exposed soil, which creates significant local surface roughness differences. Runoff is more likely to concentrate in sparsely vegetated areas, forming high-velocity runoff paths, thereby enhancing shear stress. Runoff shear stress is not only dependent on flow velocity but is also closely related to runoff depth [38]. At 60% cover, the runoff depth was relatively large, further increasing the tangential force exerted by the water on the soil. Moreover, the discontinuous vegetation distribution significantly altered runoff paths, causing runoff to rapidly concentrate in areas with sparse vegetation [19,20], forming a localized concentrated flow effect that intensified shear stress. By contrast, under bare land and 30% cover conditions, although the flow velocity was higher, the surface roughness was lower and more uniform, limiting the velocity gradient and thus resulting in relatively low shear stress [39]. At 90% cover, vegetation formed a continuous resistance layer, effectively reducing flow velocity and dispersing runoff paths, which weakened shear stress [40].

### 4.3. Effects of Different Vegetation Patterns on Hydrodynamic Parameters

**Figure 11:**
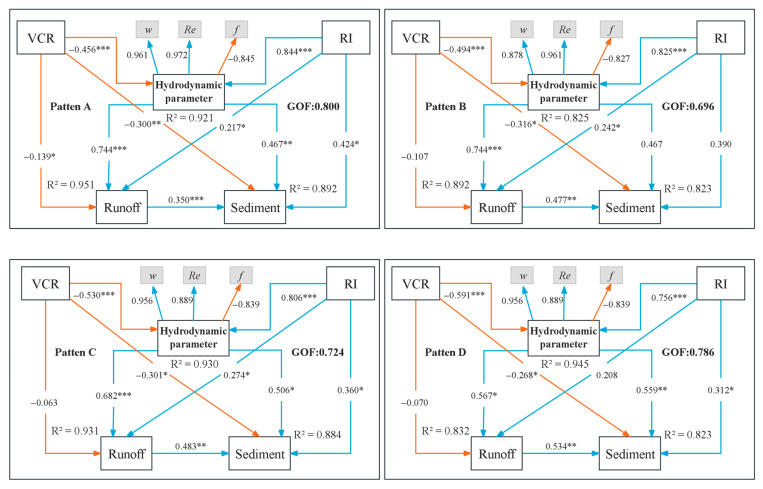
Partial least squares path patterns (PLS-PM) showing the effects of vegetation coverage and rainfall intensity on hydrodynamic parameters, runoff, and sediment yield under different vegetation patterns. RI represents rainfall intensity; VCR indicates vegetation cover; and GOF denotes the structural equation fit. The number on the arrow represents the standardized path coefficient, the blue line arrow is positive, the orange line arrow is negative, and *R^2^* represents the degree of variation explained by the pattern. The path efficiency near the arrow indicates the influence of one receiving amount on the other receiving amount. The numbers near the sub-color and confluent arrows are normalized path coefficients. An asterisk indicates a significant effect (*: *p* < 0.05, **: *p* < 0.01, ***: *p* < 0.001).

Through structural equation analysis, we found that *u*, *p*, *Re*, *Fr*, *f*, *τ*, *ω*, *n*, and *h* can all serve as hydrodynamic indicators for evaluating slope erosion processes and carbon migration (Figure 11). The fitting degree of the four understory vegetation models was GOF > 0.65, and the structural equation model could well reflect the relationship between vegetation coverage, rainfall intensity, hydrodynamic parameters, runoff, and sediment yield. Vegetation coverage was significantly negatively correlated with hydrodynamic parameters, runoff, and sediment yield, while rainfall intensity was positively correlated. From the perspective of slope hydraulics and hydrodynamics, we found that under different vegetation patterns, *ω*, *Re*, and *f* more effectively characterize soil erosion processes [41,42]. Among them, *Re* and *ω* show the highest correlations with erosion, which can be used to better explain the potential erosion risk of soil by flow [43,44]. Some studies have found that flow shear stress increases exponentially with vegetation coverage [45]. This conclusion is different from our study, possibly due to pattern D’s dispersing effects on runoff, which increased surface flow resistance. Furthermore, vegetation interception diverts flow direction down the slope, thereby reducing flow velocity [46]. Additionally, this may be due to pattern D reducing flow velocity and turbulence, thereby weakening the local effects of shear stress and reducing its role in soil erosion mechanisms [47]. Thus, in this study, flow shear stress may not be a suitable indicator for evaluating slope soil erosion. Different vegetation coverages had a significant regulatory effect on slope hydrodynamics and soil erosion. Pattern D reduced the flow velocity, Reynolds number, and runoff power, thereby mitigating slope erosion as a whole. Pattern D vegetation could not only intercept sediment through its own mechanical action but also change the slope topography and reduce the slope through sedimentation, thus effectively reducing soil erosion. Therefore, on southern red soil slopes, understory vegetation configurations should be configured with a certain vegetation coverage and combined with pattern D in order to intercept uphill soil erosion, thereby reducing the overall amount of soil erosion. In the process of land use development, it is recommended to preferentially combine pattern D to plant perennial shrubs and herbs in the understory area.

## 5. Conclusions

Vegetation cover significantly reduced soil erosion and runoff. Slope runoff and sediment yield diminished as vegetation cover increased, and an increase in vegetation cover significantly inhibited soil erosion in forests (*p* < 0.05). When vegetation cover reached 60%~90%, pattern C and pattern D showed the most pronounced effects in reducing soil erosion. The slope flow velocity showed a similar trend: as vegetation cover increased, flow stability improved. At low coverage, slope flow tended toward ‘turbulent rush flow,’ while at high coverage, flow predominantly occurred as laminar or slow flow. Patterns C and D most significantly reduced flow velocity and depth (*p* < 0.05), thereby weakening turbulence and further reducing soil erosion risk. Structural equation analysis revealed that under different vegetation cover patterns, the runoff power (*ω*), Reynolds number (*Re*), and resistance coefficient (*f*) more accurately characterized soil erosion processes. Among these, the Reynolds number and runoff power were the dominant factors affecting soil erosion on red soil slopes, while flow shear stress was significantly reduced under high-coverage conditions. Implementing vegetation coverage in segments and near the outlet, with coverage ranging from 60% to 90%, can effectively reduce soil erosion.

## Data Availability

The original contributions presented in this study are included in the article. Further inquiries can be directed to the corresponding author.
